# Societal and equity challenges for Brain Health Services. A user manual for Brain Health Services—part 6 of 6

**DOI:** 10.1186/s13195-021-00885-6

**Published:** 2021-10-11

**Authors:** Richard Milne, Daniele Altomare, Federica Ribaldi, José Luis Molinuevo, Giovanni B. Frisoni, Carol Brayne, on behalf of the European Task Force for Brain Health Services

**Affiliations:** 1 Society and Ethics Research Group, Wellcome Connecting Science, Hinxton, UK; 2 Cambridge Public Health, University of Cambridge, Cambridge, UK; 3 Laboratory of Neuroimaging of Aging (LANVIE), University of Geneva, Geneva, Switzerland; 4 Memory Clinic, Geneva University Hospitals, Geneva, Switzerland; 5 Laboratory of Alzheimer’s Neuroimaging and Epidemiology (LANE), Saint John of God Clinical Research Centre, Brescia, Italy; 6 Department of Molecular and Translational Medicine, University of Brescia, Brescia, Italy; 7 Barcelonaβeta Brain Research Center, Pasqual Maragall Foundation, Barcelona, Spain

**Keywords:** Brain health services, Dementia, Aging, Alzheimer’s disease, Prevention, Public health, Equity

## Abstract

Brain Health Services are a novel approach to the personalized prevention of dementia. In this paper, we consider how such services can best reflect their social, cultural, and economic context and, in doing so, deliver fair and equitable access to risk reduction. We present specific areas of challenge associated with the social context for dementia prevention. The first concentrates on how Brain Health Services engage with the “at-risk“ individual, recognizing the range of factors that shape an individual’s risk of dementia and the efficacy of risk reduction measures. The second emphasizes the social context of Brain Health Services themselves and their ability to provide equitable access to risk reduction. We then elaborate proposals for meeting or mitigating these challenges. We suggest that considering these challenges will enable Brain Health Services to address two fundamental questions: the balance between an individualized “high-risk” and population focus for public health prevention and the ability of services to meet ethical standards of justice and health equity.

## Background

The development and implementation of “precision” Brain Health Services (BHSs) represent a novel approach to reducing the risk of dementia in older adults. In this paper, we consider how such services can best reflect their social, cultural, and economic context and, in doing so, deliver fair and equitable access to risk reduction. Our aim is to provide a constructive critique and initiate discussion around the challenges faced and raised by brain health programs.

We focus on the model of the BHS as developed by Frisoni et al. [1]. BHSs set out a model of personalized prevention, based around individualized multidomain interventions and educational activities and, in anticipation of potential disease-modifying therapies, around reshaped secondary care services. Whereas those with objective cognitive impairment are often served by existing memory clinics, BHSs extend to those without objective cognitive impairment but are concerned they are at higher risk of developing dementia for a potentially varying set of reasons, including family history. BHSs feature specific organization, structure and challenges [2], and have four main missions (extensively discussed in the pertinent papers published in this issue of *Alzheimer’s Research & Therapy*): dementia risk profiling [3], dementia risk communication [4], dementia risk reduction [5], and cognitive enhancement [6].

Our discussion proceeds as follows. First, we consider the practical challenges associated with the social and economic context for the delivery of BHSs. In each section, we frame these around a specific challenge and suggest potential practical, policy and research responses. In closing, we argue that situating BHSs in their social context requires attention to the ability of such services to deliver access to risk reduction in a socially just and equitable fashion.

We present two specific areas of challenge associated with the social context for dementia prevention. The first concentrates on how BHSs engage with the “at-risk” individual, recognizing the range of factors that shape an individual’s risk of dementia and the efficacy of risk reduction measures. The second emphasizes the social context of BHSs themselves and their ability to provide equitable access to risk reduction. Rather than simply pointing out challenges, however, we introduce proposals for remedying or mitigating them. Considering these challenges would enable BHSs to address two fundamental questions: the balance between a “high-risk” and “population” focus for public health prevention and the ability of services to meet ethical standards of justice and health equity.

## The at-risk individual in social context

### The social determinants of dementia risk

- *Challenge*. A focus on self-referring high-risk individuals within specialist BHSs does not reflect the wider social and economic determinants of brain health and cannot be the most effective and efficient approach to risk reduction at a population level.
- *Elaboration*. An individual’s risk of developing dementia is a consequence of their genetic background and the accumulation of factors over the life course [7, 8]. Globally, it is estimated that more than a third of the incidence of dementia in the population is due to exposure to potentially modifiable risk factors. The age-specific incidence of dementia is falling in many countries as a result of improvements in childhood education, nutrition, healthcare, and lifestyle changes [9, 10].
  These findings have clear implications for the promotion of brain health. The Lancet Commission focuses recommendations on 12 modifiable risk factors: the treatment of hypertension, childhood education, exercise, social engagement, smoking and alcohol consumption, hearing loss, depression, diabetes, obesity, air pollution, and traumatic brain injury [8]. Many of these risk factors may be amenable to behavior change and lifestyle approaches. However, among them are those that are not easily incorporated into an approach to brain health focused on the individual rather than populations or groups—such as access to education in early life or exposure to air pollution. In fact, even factors which are often considered as “individually” modifiable are unequally distributed, are closely associated with wider forms of social, economic, health, and gender inequality, and are grounded in social and economic conditions that run through the life course [8, 11, 12]. Importantly, they also reflect structurally and institutionally embedded inequalities and discrimination related to race/ethnicity—both in the social distribution of risk factors, such as access to education, and the representation of diverse populations in clinical and epidemiological datasets [13, 14].
  The implications for BHSs are twofold. First, primary prevention activities in populations have already been shown to have significant societal value. Thinking how to develop these sensibly within contemporary populations would be a worthwhile focus for research and policy. Second, a “high-risk” focus needs to engage with social and economic determinants of dementia risk, and their uneven distribution across populations, as this is where the greatest gain may be achieved in terms of future risk reduction.
- *Solution*. Individualized approaches to brain health need to explicitly account for the social and economic distribution of risk across the life course in order to shift population risk profiles for cognitive decline and dementia at each life stage [15]. All BHSs should commit to programs that are not only individual risk reduction programs, but that aim to address economic and social determinants of health and disease by empowering individuals and communities who are at greatest disadvantage.

### Risk communication and behavior change

- *Challenge*. The concept of BHSs relies largely on individualized risk prediction, using models derived from a combination of lifestyle, genetic, and biomarker information. This information forms the basis for personalized “precision” prevention plans. It is essential, however, that these interventions recognize the psycho-social challenges and complexity associated with behavior change and acknowledge the uncertainties associated with risk prediction models.
- *Elaboration*. The use of genetic, biomarker, and lifestyle information to identify “high-risk” individuals for “secondary prevention” is central to the BHS model. The use of this information to make significant differences to individual risk, however, remains understudied. Here, there is potential to learn from risk reduction programs in other clinical areas. The central message of systematic reviews of this work has been that the individualized provision of risk information alone has neither strong nor consistent effects on health-related behaviors [16]. In the case of cardiovascular disease, it has been shown that personalized risk information can improve the accuracy of an individual’s perception of their risk and may have implications for improving clinical prescribing but, again, has little effect on the way individuals live their lives [17]. In the case of genetic risk information, trials of the provision of genetic risk information have shown no or very limited effects on health behaviors [18].
  For dementia itself, there is also a need to be cognizant of the uncertainties associated with risk information. These uncertainties reflect the limitations of the evidence base that informs current prediction models when applied to individuals and have consequences for those acting upon them [19]. Thus, for example, there is evidence of differing effects of ancestry on genetic risk but also significant under-representation of racially and ethnically diverse populations in studies of imaging and genomics, as well as clinical trials [20–23]. Uncertainties are likely to be particularly exacerbated among those attending BHSs from the general population, rather than traditional memory clinic populations or among those with a diagnosis of mild cognitive impairment [24–26].
  Sub-analyses of the FINGER and MAPT studies suggest that a multimodal programs of risk reduction may have some, limited in size, value for high-risk individuals, particularly those at greater genetic risk (i.e., *APOE* ɛ4 carriers) [27] or with brain amyloidosis [28]. However, these were not the primary endpoints and such findings must be confirmed in targeted studies. Together, the current risk reduction interventions and the apparently limited value of approaches based solely on risk communication suggest there is an opportunity for work on dementia prevention to learn from, rather than repeat, the mistakes of other fields. To make a significant difference, BHSs should have a robust model of the complexity of health behaviors and their change [29].
- *Solution*. Approaches to risk reduction that rely on individualized risk prediction leading to behavior changes are likely to be of limited effectiveness. Indeed, the vast bulk of evidence about changing behavior takes account of the fact that much decision making is automatic, and that changing behavior involves a combination of capability, motivation, and opportunity [30]. This may include changes to the environment to enhance the capacity for healthy behaviors to occur. For example, there is some evidence that approaches to CVD risk reduction focusing on a range of community based approaches may not only be efficacious but also reduce inequalities in health outcomes [31, 32]. In the case of dementia prevention, this may include the creation of life-course environments that encourage physical activity, good nutrition, educational opportunities, social engagement, and a healthy physical environment.
  In engaging with uncertainty, the actual nature of the evidence base, rather than the hyperbole, for prediction models and individualized risk reduction programs should be discussed with members of the public and patients who seek such approaches, in relation to their age, gender, and race/ethnicity, with regard to short-, medium-, and long-term prospects of benefit, known harms, and likely cost.

## The BHSs in a social context

### Access to BHSs

- *Challenge*. Specialist BHSs risk compounding issues of access associated with existing clinical services, limiting their value to disadvantaged populations.
- *Elaboration*. Expanding services targeting the “worried well” risk reinforcing the “inverse care law,” targeting those already well-served by health care and preventative health services, many of whom would probably have relatively little likelihood of benefitting and are probably most likely to have optimal brain health [33]. Where these services are reimbursed, it is important to consider the trade-offs with services that are not being implemented, with better evidence bases that might achieve more for more people [34]. Where private health care providers offer services, potential customers who feel they may be beneficial should be informed about the state of evidence and its limitations. The recent history of direct-to-consumer genetic testing and “brain training” tools shows that there are likely to be many products and services whose marketing may lead individuals to assume a health benefit that is not justified by the evidence base [35, 36].
  In addition, interventions that require an individual to draw on their own resources—whether social, economic, or psychological—tend to disproportionately benefit those with more of these resources [37]. This can result in the accumulation of disadvantage—both through exposure to risk, as discussed above, and in terms of access to testing and diagnostic services.
  Access to memory clinics, and even dementia services, is unequally distributed within and between countries, and is low around the world, varying significantly by country. Some such inequalities are a result of the geographical distribution of services within countries, particularly where they are concentrated in urban areas [38, 39]. People living in rural areas may thus have less access to specialist dementia services and post-diagnostic support, either because they do not exist or are inaccessible by public transport, and there may be a greater emphasis on the role of primary care [40].
  Further, in the UK and USA, access to a diagnosis has been shown to be associated with older age, male gender, and higher level of education and socio-economic status including income [41–43]. Those from less deprived socio-economic groups are more likely to be initiated on anti-dementia drugs than the most deprived and may also present at clinic earlier in the progression of symptoms [44, 45]. Race/ethnicity is also associated with access. In the USA, those from non-Hispanic Black and Hispanic populations are more likely to have a missed or delayed diagnosis, less likely to access diagnostic services later, and be less likely to access medication or research trials [46–48]. Similar findings have been found in the UK for Asian men and women, and Black men [43, 49, 50]. Importantly though, data on race/ethnicity is often not collected—in a study based on a representative sample of UK primary care records, race/ethnicity was far more likely to be missing from records than information on deprivation [49]. These lacunae in the data present challenges in understanding who is accessing, and who is excluded, from BHSs, and further exacerbate the biases in data discussed above.
  Further, access to BHSs is likely to be shaped by cultural framings of both dementia and risk. Available evidence suggests cultural factors that shape access to memory clinics, and, while specific evidence is lacking, it seems likely they would similarly affect the distribution of those who attend BHSs. Such cultural factors may differ by country and between racial or ethnic groups. Qualitative research, for example, suggests potential cultural differences related to the recognition or acceptance of dementia, or hesitations in seeking a diagnosis among minority ethnic groups, such that medical help may not be sought and dementia seen as a private problem, associated with significant social stigma [51–54]. In the USA, public expectations related to learning dementia risk may also reflect concerns about employment and insurance discrimination, considerations that, as Frisoni et al. rightly point out, impact on the decision to communicate risk status. Such considerations also shape how people access services [55].
  To ensure that BHSs address and do not exacerbate inequalities in access, we need robust trial evidence before extending reimbursed BHSs to asymptomatic individuals. The overall benefit to societies of targeted early detection services has not yet been proven in clinical trials and the only international study of screening did not reveal any tangible benefits [34]. It is important to acknowledge the existing clinical context for memory clinics that themselves were not developed out of an evidence base but out of a need to identify individuals at an earlier stage of dementia or cognitive impairment from which to recruit to trials.
  Here again, the development of the BHSs might usefully draw on the experience of other areas, such as the introduction of routine health checks in the field of cardiovascular disease prevention. The introduction of general health checks for reducing illness or mortality has been evaluated as unlikely to be beneficial [56]. In fact, both health checks and individualized behavior change approaches may have potentially negative impacts on overall population health equity [37, 57]. In contrast, evaluations of interventions in healthy eating suggest that those that focus “upstream,” for example by intervening in price, are likely to reduce inequalities [32]. The development of brain health programs should consider and evaluate the impact of such programs on health inequalities and the potential for alternative approaches that may reduce inequity. This offers the opportunity to better understanding the impact of interventions across social inequalities and to increase the potential for genuinely beneficial interventions that consider whole systems and the complex realities of public health [58]. For example, modeling incorporating an explicit emphasis on equity suggests that targeting health checks to areas of disadvantage—using health care records to identify those at greatest risk of adverse outcomes—may make them more efficacious, beneficial and cost-effective [57].
- *Solution*. To be of value, investment or reimbursement for individualized approaches to risk reduction must evaluate the value for those they represent in terms of long-term brain health, where/whether an individualized approach will lead to sufficient risk reduction to be systematically supported, and what the implications are in terms of equity. This includes the routine collection and evaluation of socio-economic data related to the impact of BHSs. It also involves the exploration of interventions whose ambition is to address systemic and population-level challenges and approaches to delivery that engage with the cultural, religious, ethnic, and racial diversity of the populations they serve.

## Achieving just brain health

In the preceding sections, we have suggested that the challenges for BHSs, when considered in their social context, are considerable but not insurmountable. These challenges, and the remedies we have proposed, are summarized in Table 1. In closing, we consider the benefits of addressing them.

**Table 1:** The societal challenges encountered by the Brain Health Service model and accompanying recommendations

Challenge	Recommendation
A focus on self-referring individuals does not reflect social determinants of brain health	Individualized approaches should explicitly account for social and economic distribution of risk Brain Health Services should commit to wider work with communities to address social and economic determinants of health
Individualized risk reduction strategies face significant psycho-social barriers to implementation	Measures to reduce risk should recognize the importance of changing environments, rather than behaviors The evidence for risk reduction should be discussed with individuals contemplating changing behaviors
Specialist Brain Health Services risk compounding inequalities in access to clinical services	Evidence is needed of the value of extending services to asymptomatic populations in terms of long-term effects on brain health Brain health programs should consider and evaluate their impact on health inequalities

The first relates to the balance between individualized “high risk” and population prevention strategies. The former may have more significant impacts on individuals, whereas the latter may have more modest individual impacts but a greater impact across the population. Further, shifting the norm of behaviors across the population may have a subsequent impact on what is considered as “high-risk” behavior. Thus, high-risk individuals may be more likely to take physical exercise or reduce smoking if this is considered to be normal [59]. As Frisoni et al. note, following Rose, high-risk and population strategies are not inherently exclusive [1, 60]. The benefits of population prevention may sit alongside those targeting individuals with pathological changes associated with increased risk of dementia. However, as Rose also recognized, the complementarity of these approaches relies on a lack of competition for resources. In the current environment for healthcare where competition for resources is intense, a resource intensive high-risk approach inevitably limits the possibilities for population measures—even when the overall benefit of the latter may be greater.

The development of BHSs thus requires addressing a wider range of issues than currently considered within the “ethics” of BHSs. Discussion of the ethics of dementia prevention has been dominated by a focus on autonomy, particularly the right of individuals to know, or not to know, their risk [1, 61]. The challenges presented here emphasize the critical and compelling importance of widening this discussion, particularly as the concerns of the memory clinic and clinical ethics encounter those of population health, public health, and societal ethics. It has been recognized that it is essential that efforts to prevent dementia “leave no one behind” [62]. Social justice must be embedded as a core value and guiding principle for brain health programs [63, 64]. This means improving health to improve well-being, by focusing on the needs of the most disadvantaged, to ensure the fair distribution of common advantages and the sharing of common burdens. At heart, following Beauchamp’s framing of public health ethics, it requires thinking about and reacting to the problem of brain health as “primarily collective problems of the entire society” [64].

## Conclusions

BHSs that focus on the delivery of personalized risk scores and interventions targeted at “high risk” individuals may have potential as an approach limited to reducing risk in some sections of the population. However, this must be developed hand in glove with improving the means of reducing the overall population burden of dementia through life-course and broader societal measures. It is these measures that will change the incidence and prevalence of dementia, as well illustrated in the last 50 years. It is therefore essential that those who invest in the development of BHSs aimed at individualized services consider the just allocation of resources between approaches. While high-risk and population approaches can be complementary, they risk competing for scarce resources, particularly given the resource-intensiveness of clinical risk assessment and follow-up. Without attention to the factors discussed here, an approach focusing primarily on high-risk populations is likely to struggle to deliver fair and equitable access to services or to risk reduction.

## Data Availability

Data sharing is not applicable to this article as no datasets were generated or analyzed during the current study.

## Abbreviation

BHS: Brain Health Service
